# Müller cell activation, proliferation and migration following laser injury

**Published:** 2009-09-17

**Authors:** Mark A. Tackenberg, Budd A. Tucker, Jesse S. Swift, Caihui Jiang, Stephen Redenti, Kenneth P. Greenberg, John G. Flannery, Andreas Reichenbach, Michael J. Young

**Affiliations:** 1 Schepens Eye Research Institute, Department of Ophthalmology, Harvard Medical School, Boston, MA; 2 Paul-Flechsig Institute of Brain Research, Faculty of Medicine, University of Leipzig, Leipzig, Germany; 3 Interdisciplinary Center of Clinical Research (IZKF) Leipzig, Faculty of Medicine, University of Leipzig, Leipzig, Germany; 4 Helen Wills Neuroscience Institute, University of California, Berkeley, CA; 5 MCB Neurobiology, University of California, Berkeley, CA

## Abstract

**Purpose:**

Müller cells are well known for their critical role in normal retinal structure and function, but their reaction to retinal injury and subsequent role in retinal remodeling is less well characterized. In this study we used a mouse model of retinal laser photocoagulation to examine injury-induced Müller glial reaction, and determine how this reaction was related to injury-induced retinal regeneration and cellular repopulation.

**Methods:**

Experiments were performed on 3–4-week-old C57BL/6 mice. Retinal laser photocoagulation was used to induce small, circumscribed injuries; these were principally confined to the outer nuclear layer, and surrounded by apparently healthy retinal tissue. Western blotting and immunohistochemical analyses were used to determine the level and location of protein expression. Live cell imaging of green fluorescent protein (GFP)-infected Müller cells (AAV-GFAP-GFP) were used to identify the rate and location of retinal Müller cell nuclear migration.

**Results:**

Upon injury, Müller cells directly at the burn site become reactive, as evidenced by increased expression of the intermediate filament proteins glial fibrillary acidic protein (GFAP) and nestin. These reactive cells re-enter the cell cycle as shown by expression of the markers Cyclin D1 and D3, and their nuclei begin to migrate toward the injury site at a rate of approximately 12 μm/hr. However, unlike other reports, evidence for Müller cell transdifferentiation was not identified in this model.

**Conclusions:**

Retinal laser photocoagulation is capable of stimulating a significant glial reaction, marked by activation of cell cycle progression and retinal reorganization, but is not capable of stimulating cellular transdifferentiation or neurogenesis.

## Introduction

Müller cells, the major glial cell type in the mammalian retina, were once considered to be nothing more than the “glue” between retinal neurons. The name “glia” is derived from the Greek word for glue. In recent years, many studies have shown that Müller glia function as more than mere structural support. They have been demonstrated to play essential roles in maintaining homeostasis in the inner retina, clearing the extracellular space of “used” neurotransmitters, aiding in forming the blood-retinal barrier, protecting retinal neurons against free radicals, providing metabolic support, and even transmitting light through the retina by acting as optical fibers [1-6]. Furthermore, recent studies have suggested that Müller cells are capable of re-entering the cell cycle, dedifferentiating, adopting certain characteristics of progenitor and stem cells that migrate to the damaged retinal tissue, and producing new neurons following specific types of retinal injury (a process collectively known as transdifferentiation) [7-9]. Driven by these findings, various groups have begun to investigate ways to increase the regenerative capacity of these glial cells through the use of molecules such as sonic hedgehog (shh), wnt, and notch, as they play important roles in developing nervous tissue and are in part responsible for determining cell fate [10-12]. Although studies claim to have successfully increased the number of Müller cells expressing neuronal progenitor cell markers following administration of a pan-retinal toxin [13], there is limited information on the ability of these cells to respond to other types of injury by undergoing transdifferentiation and directed migration. As such, in the present study, we have focused on the use of retinal laser photocoagulation to study the responses of local Müller glia to a defined local mode of injury.

Retinal photocoagulation is FDA-approved and currently in use to treat several severe degenerative (diabetic retinopathy) and acute (retinal detachment) human eye diseases [14]. More importantly for the present study, laser photocoagulation produces a local circumscribed area of damage, surrounded by relatively healthy tissue, which allows us to study Müller cell behavior, including the ability to undergo transdifferentation without affecting the entire retina. It is hypothesized that retinal injury, induced by laser photocoagulation, will stimulate Müller glia cycle progression, dedifferentiation, stem cell marker expression, and cellular transdifferentation.

## Methods

### Animals

C57BL/6 mice were obtained from and maintained in the animal facility of the Schepens Eye Research Institute and used at the age of two to six months, with the exception of the live imaging experiments, which included controls of age postnatal day 1–10. Animals were maintained in standard cages with a 12:12 light dark cycle and provided water and food ad libitum. All experimental procedures were approved by the Institute’s Animal Care and Use Committee and adhered to the ARVO Statement for the Use of Animals in Ophthalmic and Vision Research. Experimental groups consisted of n=5 for each time point of all experiments.

### Laser photocoagulation and euthanasia

A diode laser at a wavelength of 810 nm was used to create 12 laser burns per eye (three per quadrant) at the region of the posterior pole. Each burn was 350 µm in diameter and produced with 100 mW of power for 100 ms. Spot size, laser duration, and laser intensity were chosen based on the normal range of FDA-approved treatments used to treat diseases such as diabetic retinopathy and diabetic macular edema [15-18]. Mice were euthanized after one through seven days post-laser burn by CO_2_ inhalation.

### Histology and immunohistochemistry

Eyes were enucleated, fixed with 4% paraformaldahyde (PFA) for 24 h, cryoprotected in sucrose (10% and 30% sucrose in distilled water [DW], 24 h each), and embedded in optical coherence tomography (OCT; Sakura Finetek, Torrence, CA). Tissue was then frozen on dry ice and sliced into 16 µm sections using a Minotome Plus cryostat (Triangle Biomedical Sciences, Durham, NC). Sections were permeabilized using 0.1% Triton X-100 in 10% goat serum (or 3% bovine serum albumin [BSA]) for 1 h. Samples were incubated overnight (12 h) at 4 °C with primary antibodies directed against glial cell markers, which included 1:250 glial fibrillary acid protein (GFAP, monoclonal), 1:250 glutamine synthetase (GS, monoclonal; Chemicon, Billerica, MA), 1:250 vimentin (polyclonal; Sigma, St. Louis, MO), 1:250 cellular retinaldehyde-binding protein (CralBP, monoclonal; Abcam, Cambridge, MA), and 1:100 Cyclin D3 (polyclonal; Santa Cruz, Santa Cruz, CA). The activated Müller cell markers used included 1:250 nestin (monoclonal) and 1:100 PAX-6 (monoclonal; Chemicon). We also used the cell cycle marker 1:250 Cyclin D1 (polyclonal; NeoMarkers, Fremont, CA), and progenitor and stem cell markers, which included 1:250 SOX-2 (monoclonal), 1:100 MASH-1 (monoclonal; R&D Systems, Minneapolis, MN), 1:200 OTX-2 (monoclonal; Abcam), and 1:100 CHX-10 (polyclonal; Chemicon). Samples were then rinsed 3×10 min in PBS (8 g of NaCl, 0.2 g of KCl, 1.44 g of Na_2_HPO_4_, 0.24 g of KH_2_PO_4_) and incubated with either Cy2 or Cy3- conjugated secondary antibodies for 1–2 h (Jackson ImmunoResearch, West Grove, PA) at room temperature. Finally, samples were rinsed for 3×10 min in PBS and sealed in mounting media (Vector Laboratories, Burlingame, CA) for imaging, using a Leica confocal microscope.

### Müller cell virus preparation

The AAV8-mGFAP-eGFP vector was packaged in AAV293 (Stratagene, La Jolla, CA) cell cultures grown in standard growth media that contained the following ingredients: Dulbecco’s Modified Eagle Medium (DMEM), 10% heat inactivated fetal bovine serum (FBS), and 4 mM L-glutamine. Cells were transfected with three plasmids using Lipofectamine 2000 transfection reagent (Invitrogen, Carlsbad, CA). The three plasmids used in the transfection were: 1) Ad helper plasmid; 2) AAV helper; and 3) the AAV transfer vector containing the mouse GFAP promoter followed by the enhanced green fluorescent protein (eGFP) cDNA, and the woodchuck hepatitis virus post-transcriptional regulatory element (WPRE) flanked by AAV2 inverted terminal repeats. The mouse GFAP promoter was subcloned from the vector pFmGFAPGW previously described in detail [19]. The virus was isolated from AAV293 cells through three freeze–thaw cycles. It was then incubated for 30 min at 37 °C with 50 U benzonase (Novagen, Madison, WI) and further purified by iodixanol density gradient centrifugation, buffer exchange, and concentration as previously reported [20]. The titer in vector genomes/ml (vg/ml) of final virus isolate was determined by a quantitative real time-polymerase chain reaction.

### Live imaging

Mouse eyes were injected subretinally with 1 μl of a Müller cell specific adeno-associated vector, containing a GFP tag. After two weeks, these eyes underwent laser injury and were then enucleated two to three days later. The retina was isolated and securely attached to a membrane filter (Whatman, Dassel, Germany) with the photoreceptor layer facing down (the membrane filter was used to stabilize the tissue and prevent movement when imaging), cut into 120 μm-thick sections, and mechanically stabilized in a perfusing chamber with the cut side up. The tissue was constantly perfused with an extracellular solution that consisted of the following ingredients: 110 mM NaCl, 3 mM KCl, 2 mM CaCl_2_, 1 mM MgCl_2_, 1 mM Na_2_HPO_4_, 0.25 mM glutamine, 10 mM N-2-hydroxyethylpiperazine-N’-2-ethanesulfonic acid (HEPES), 11 mM glucose, and 25 mM NaHCO_3_, adjusted to pH 7.4 with Tris-(hydroxymethyl) aminomethane (Tris-base) and bubbled with carbogen (95% O_2_, 5% CO_2_). The slices were then imaged every 15 min with a Leica confocal microscope for 3 h, and cell movement was measured by creating and comparing projections of each time point using the Leica confocal imaging software. As the preparation procedure used for live imaging itself may stimulate additional cues for Müller cell migration, we were careful to ensure that the site of retinal laser burn was located in the center, away from the cut edges of the retinal section being imaged. Unlike using a standard epi-fluorescence setup, which would require thin tissue sections through the injury site to achieve a focused image, the confocal microscope allowed us to zoom into the tissue directly on the laser injury without disruption of surrounding structures.

### Western blotting

For western blot analysis, retinas from laser-burned eyes were homogenized in lysis buffer: 50 mM Tris-HCl, pH 7.6, 150 mM NaCl, 10 mM CaCl_2_, 0.1% Triton X-100, and 0.02% NaH_3_. The mixture was centrifuged, supernatants were isolated, and protein concentrations were determined using a bicinchoninic acid (BCA) protein assay (Pierce Chemical, Rockford, IL). Equivalent amounts of protein (50 µg) were subjected to SDS–PAGE (8%–10% acrylamide), transferred to nitrocellulose, and probed with the following antibodies: 1:1,000 GFAP (monoclonal), 1:1,000 GS (monoclonal) and 1:1,000 PAX-6 (monoclonal; Chemicon), 1:1,000 Cral-BP (monoclonal), 1:1,000 vimentin (polyclonal), 1:1,000 beta-actin (monoclonal; Abcam), 1:1,000 Cyclin D1 (polyclonal; Thermo Scientific, Wyman, MA), 1:1,000 Cyclin D3 (polyclonal; Santa Cruz), 1:1,000 nestin (monoclonal; DSHB, Iowa City, IA), and 1:1,000 SOX-2 (monoclonal; R&D Systems). Blots were cut and reprobed sequentially, visualized with ECL reagents (NEN, Boston, MA), and exposed to X-ray film (Kodak/Carestream Health, Bio Max Light Film, Rochester, NY). Developed films were subsequentially digitized and densitometrically analyzed with ImageJ software (National Institutes of Health, Washington, DC). Digital images of western blots were used to make composite figures with graphics software (Adobe Photoshop, Adobe Corp., San Jose, CA).

### Statistical analysis

The data were plotted as mean±standard deviation, with significance 161 noted only if p≤0.05, as determined by an unpaired *t*-test. Asterisks denote the following levels of confidence: *≤0.05, **≤0.01, and ***≤0.001.

## Results

### Laser burn-induced Müller cell activation and cell cycle progression

**Figure 1:**
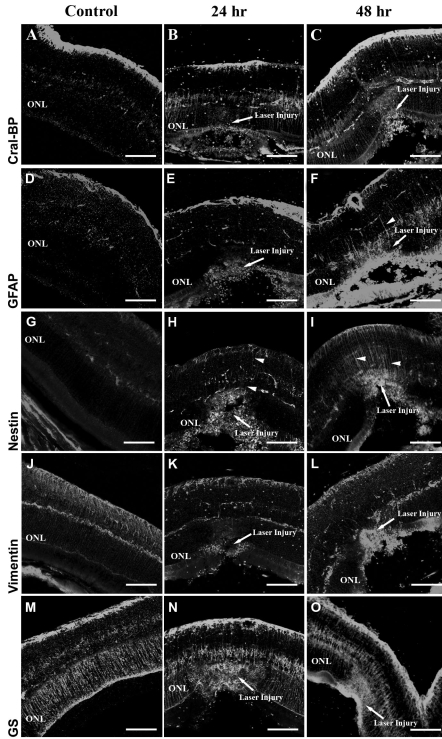
Müller cell activation following laser burn injury. Following retinal laser burn, eyes were enucleated, cryosectioned, and immunostained using the glial markers Cral-BP (**A**-**C**), GFAP (**D**-**F**), nestin (**G**-**I**), vimentin (**J**-**L**), and GS (**M**-**O**). Cral-BP was upregulated at 24 and 48 h within the injury site (**A**-**C)**. GFAP was upregulated at 48 h postinjury (**F**). Nestin was upregulated at 24 h and 48 h (**G**-**I**) postinjury. Vimentin and GS did not show a significant increase in expression at any time point (**J**-**O**). The scale bar represents 75 μm. Abbreviation: outer nuclear layer (ONL). Arrowheads indicate Muller cell processes.

To study the glial response to retinal laser injury, we performed immunohistochemical analysis for the glial cell markers GS, Cral-BP, GFAP, nestin, and vimentin. Cral-BP, evident in the normal retina, was upregulated within 24 h of laser burn and stayed elevated for at least 48 h postinjury (Figure 1A-C). Unlike Cral-BP, GFAP was not detectable in Müller cells within the normal retina, and was only seen at 48 h postinjury (Figure 1D-F, presence of GFAP is indicated by positive staining of cellular processes in Figure 1F, arrowheads). Nestin was upregulated within 24 h after injury and increased further by 48 h (Figure 1G-I). Like GFAP, nestin was most prominent in Müller cell processes directly adjacent to the laser burn (Figure 1H,I arrowheads). Unlike the markers mentioned above, expression of both vimentin and GS were detected in control-uninjured animals and maintained at a similar level across all time points (Figure 1J-O). This suggests that unlike nestin, GFAP and Cral-BP, vimentin, and GS are not indicative of Müller cell activation.

**Figure 2:**
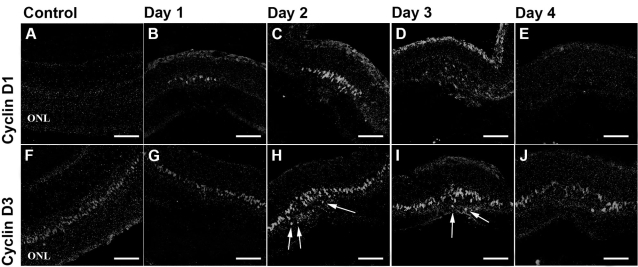
Expression of cell cycle markers. Cell bodies in the inner nuclear layer (INL) reentered the cell cycle, began to proliferate, and migrated to the ONL following laser injury. The cell cycle marker Cyclin D1 and the Müller cell nuclear marker and cell cycle marker Cyclin D3 were used to identify proliferative cells. Cyclin D1 staining was elevated within 24 h after laser injury and localized within the injury site only. Cyclin D3 expression was increased at days 2 and 3 postinjury (**H**,**I**). Positive cells normally found in the INL were now located in the outer nuclear layer, indicated by arrows **H** and **I** (ONL; **H**-**J**). The scale bars represent 75 μm.

The cyclins are key regulators of the cell cycle and are therefore upregulated in proliferative cells. Cyclin D1, upregulated in the G_1_-phase of the cell cycle, could be found within 24 h after laser burn at the injury site only and remained upregulated until three days postinjury (Figure 2A-E). Cyclin D3 is closely related to Cyclin D1 and also expressed in the G1 phase of the cell cycle. However, this molecule has been shown to also act as a normal Müller cell nuclear marker and as such can be used to track Müller cell movement and nuclear location [21]. Cyclin D3 showed slight increases in expression at both 2 and 3 days post injury (Figure 2F-J). Interestingly, some of the Müller cell nuclei that stain positive for Cyclin D3 were found to be located within the injured outer nuclear layer (Figure 2H,I). As shown in other injury models, these findings suggest that Müller cells have the potential to migrate away from their original location toward the sight of retinal injury.

**Figure 3:**
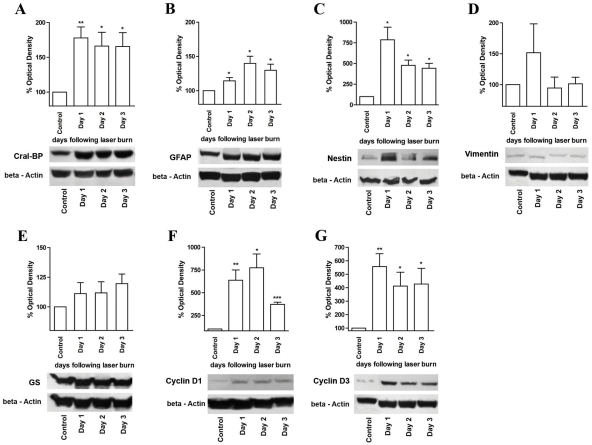
Western blot analysis of Müller cell- and cell cycle marker expression. Western blots were performed and analyzed to confirm our immunohistochemical findings. Antibodies, targeted against Cral-BP (**A**), GFAP (**B**), nestin (**C**), vimentin (**D**), GS (**E**), Cyclin D1 (**F**), and Cyclin D3 (**G**), were used. A significant increase in the expression of the markers Cral-BP, GFAP, nestin, Cyclin D1, and Cyclin D3 was observed. Asterisks denote the following levels of confidence: *≤0.05, **≤0.01, and ***≤0.001.

To further confirm our immunohistochemical results, we performed western blotting analysis of lysates isolated from laser-injured retina taken at times 0 through day 3. Although slight alterations and elevations in the levels of GS and vimentin expression were detected, these changes were not statistically significant (Figure 3E,D). Cral-BP and nestin were upregulated significantly at day 1 following laser burn and remained elevated above control throughout the remaining time points (Figure 3A,C). Although a significant increase in GFAP expression was not detected immunocytochemically until 48 h postinjury, a significant increase in this marker was detected at 24 h following laser burn when western blot analysis was used (Figure 3B). This could either be due to increased sensitivity of this analysis as compared to immunostaining, or because retinal astrocytes also express this marker.

As demonstrated in Figure 2, expression of the cell cycle marker Cyclin D1 and the cell cycle-Müller cell marker Cyclin D3 were both upregulated one day following laser burn (Figure 3F,G). More specifically, both markers showed at least a fourfold increase in expression that was maintained across all of the time points tested.

To determine if retinal laser injury was inducing progenitor and developmental marker expression, we performed immunohistochemical analysis directed against SOX-2, CHX-10, OTX-2, and MASH-1. These experiments were performed at days 1 through 3 following laser injury, with postnatal pups P1 through P10 used as positive controls, and adult uninjured mice as negative controls. SOX-2 and CHX-10 are transcription factors expressed by a variety of developing retinal cell types, whereas OTX-2 and MASH-1 have been shown to be characteristic for developing photoreceptors [22-26]. Although SOX-2, CHX-10, OTX-2, and MASH-1 could all be detected in developing retina, their presence was not identified either before or after laser injury at any of the studied time points (data not shown). This suggests that, at least in this model, Müller cell transdifferentiation or induction of endogenous stem and progenitor cells did not occur.

### Laser injury induced migration of Muller cell nuclei

**Figure 4:**
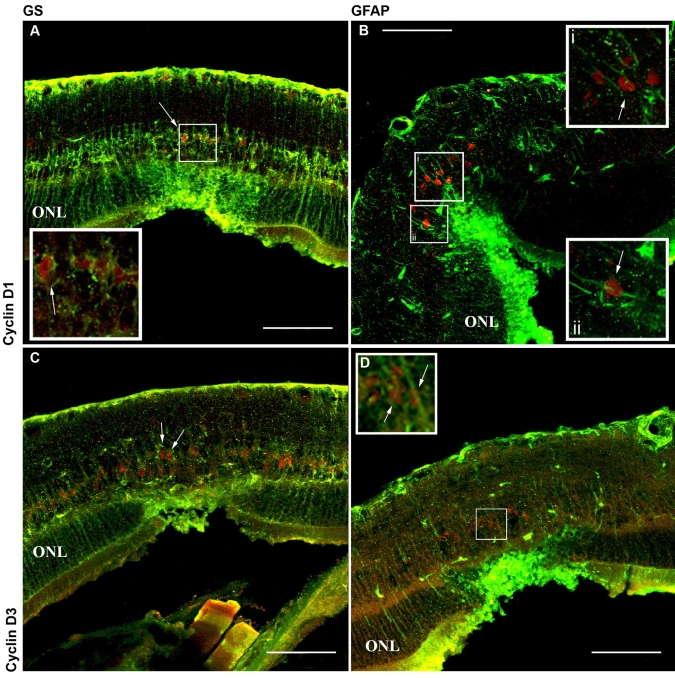
Laser injury stimulates Müller glia proliferation. Antibodies, targeted against GS (**A**, **C**; green), GFAP (**B**, **D**; green), Cyclin D1 (**A**, **B**; red), and Cyclin D3 (**C**, **D**; red) were applied at two days postretinal injury. Müller glia within the injury site stain positive for the cell cycle markers Cyclin D1 and D3, indicating that laser burn induces a reentry into the cell cycle and subsequent glial cell proliferation. The scale bar represents 75 μm. Abbreviation: outer nuclear layer (ONL). Insets are higher power views of the indicated region. Arrows point to positively labeled nuclei in **A**, **B **i, and ii, **C,** and **D.**

**Figure 5:**
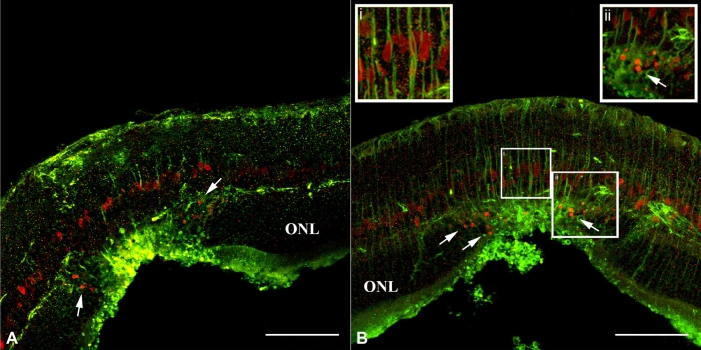
Cyclin-D3 positive cell bodies in the ONL suggested Müller cell migration. Representative examples of Cyclin D3-positive cell bodies in the outer nuclear layer (ONL) at two days following laser burn injury, suggesting possible Müller cell migration. **A**: The injury site is shown at day 2 post injury, stained with vimentin (green) and Cyclin D3 (red). **B**: The injury site is shown at day 2 post injury, stained with nestin (green) and Cyclin D3 (red). Arrows indicate Cyclin D3-positive cell bodies in the ONL localized around the injury site. The scale bar represents 75 μm. Insets are higher power views of the indicated region. Abbreviations: GCL represents ganglion cell layer.

To determine if the proliferative cells identified were truly Müller glia, we performed double labeling at days 1 through 4 following laser injury, using antibodies directed against GS (Figure 4A,C) and GFAP (Figure 4B,D), Cyclin D1 (Figure 4A,B), and Cyclin D3 (Figure 4C,D). At two days following laser burn injury, cells were detected in the inner nuclear layer that stained positive for GS and Cyclin D1 (Figure 4A), GS, and Cyclin D3 (Figure 4C), GFAP and Cyclin D1 (Figure 4B), and GFAP and Cyclin D3 (Figure 4D). This indicates that retinal laser injury stimulates Müller cell activation, cell cycle progression and subsequent proliferation. Further analysis performed two to three days after the laser burn identified the presence of cell bodies positive for Cyclin D3 located in the outer nuclear layer within the injured area of the retina only (Figure 5A,B and ii arrows). As in Figure 2, these results are indicative of Müller cell activation and subsequent injury-induced migration. However, it is possible that instead of stimulating active injury-induced migration, laser photocoagulation may be leading to passive cellular displacement. To determine whether retinal laser injury can truly stimulate active Müller cell migration, a series of live imaging experiments were performed. To carry out this analysis, Müller cell labeling via in vivo injection of a GFAP-GFP-tagged adeno-associated virus was performed. AAV-serotype 8 specifically labels Müller glia when used at the specific concentration and injection location we employed.

**Figure 6:**
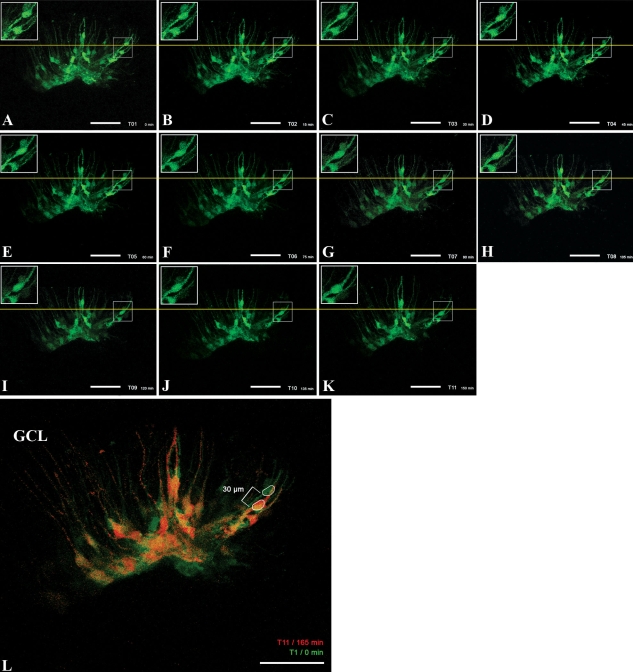
Laser injury stimulates directed Müller cell body migration. GFP-positive Müller cells were imaged for 150 min following laser burn injury. **A**-**K** shows the same area every 15 min at day 2 post injury. **L** is an overlay of the first (0 min, green) and the last (150 min, red) time point to present a comparison of the position of the cell bodies directly. The live imaging data indicated that the cell bodies of selected Müller cells were translocated 30 μm toward the injury site within those 150 min of imaging, suggesting that Müller cell bodies are able to migrate with a speed of approximately 12 μm/h. The scale bar represents 50 μm. Abbreviations: GCL represents ganglion cell layer.

Live cell imaging revealed that a portion of the Müller cell nuclei within the injury site started migrating within the outer processes of individual cells toward the outer nuclear layer at approximately two days postinjury (note that in non-injury controls, similar translocation events were not detected). Of these, Müller cell nuclei were identified that traveled upward of 30 μm over the 150 min time span imaged, indicating that a migration rate of approximately 12 μm/h can be achieved (Figure 6A-L). The distance traveled was determined by measuring from the leading edge of the cell body indicated above the yellow line in Figure 6A to the leading edge of the same cell body below the line in Figure 6K. All measurements were performed using NIHimage/Image J software. These results correspond with the aforementioned immunocytochemical data and indicate that active injury-induced migration occurs following retinal laser burn. Unlike other cell types it should be noted that Müller glia are positioned within the retina via connections formed at the inner and outer limiting membranes. Although the outer limiting membrane has been disrupted during laser injury in these studies, the inner limiting membrane appears to remain intact; as such the migratory behavior identified here is predominantly manifested as movement of the Müller glial cell nuclei within its radial process (Figure 6).

## Discussion

Müller cells play an important role in the maintenance of normal retinal structure and function. However, their responses to pathological conditions, such as those induced by laser photocoagulation, are poorly understood. Injury in many central nervous system locations is known to stimulate a “triple glial reaction,” consisting of cellular hypertrophy, cellular proliferation, and targeted cellular migration. Moreover, many groups have suggested that activation of glial cells, particularly of Müller glia, may be an initial regenerative response to injury. More specifically, it has been suggested that injury-induced Müller glial activation may encourage Müller cells to adopt stem cell characteristics, providing an endogenous source of new functioning cell types that can be integrated into damaged areas of the retina [9,13,27]. In the case of laser photocoagulation, this would require the capability of Müller cell bodies to migrate away from their original location in the inner nuclear layer, across the outer plexiform layer, and eventually into the outer nuclear layer of the retina at the site of the photoreceptor cell somata. Although similar reports of injury-induced Müller cell migration have been made (based on identification of displaced Müller glia at discreet points in time) [7,9,13], detection of this process in real time had not been performed. Thus, this study was aimed at tracking Müller cell responses to an acute localized retinal injury (induced by laser photocoagulation), in an attempt to identify the steps of injury-induced Müller cell activation including targeted migration and the potential for cellular transdifferentiation and neurogenesis.

As expected, we were able to show that retinal laser injury stimulates Müller cell activation as indicated by upregulation of the intermediate filament proteins GFAP and nestin. For instance, increased GFAP expression was detected as early as 48 h post- injury and was confined to the area of retinal laser burn. Interestingly, Müller cell activation coincided with cell cycle progression, as revealed by a local upregulation of the cell cycle marker Cyclin D1 and the cell cycle and Müller cell marker Cyclin D3. These findings are in accordance with findings from Kohno et al., who reported similar expression of both Cyclin D1 and Ki67 within the rat retina post laser injury [28]. This injury induced Muller cell proliferation could potentially provide a larger pool of cells to be employed in the process of retinal regeneration, including enhanced phagocytosis of cellular debris, restoration of retinal homeostasis, biochemical support of neuronal process (re-) growth, and, potentially, generation of new lost cell types [29]. Finally, we were able to show via live cell imaging of *AAV/GFP*-infected Müller cells that laser injury stimulates migration of activated Müller cell nuclei within its own radial processes. This migration was found to occur at a speed of approximately 12 μm/h and directed toward the injury site. A wide range of migration speeds for various mammalian cell types have been reported-i.e., astrocytes travel roughly15 μm/h [30], cerebeller granular neurons go approximately 9.6–16 μm/h [31], and oligodendrocyte precursor cells move roughly 40 μm/h [32]); these cells typically move as a complete unit from one location to another. Although the rate of migration identified here is similar to that of the aforementioned cell types, the type of migration that we have identified is more akin to that of nuclear translocation within neurogenic radial glia during cortical development. For instance, during cortical neurogenesis radial glia (a cell type which, much like Müller cells, extend processes between both basal and apical surfaces of the developing organ), undergo a series of nuclear movements known as interkinetic migration [33]. During this process, nuclear location is indicative of the stage of cellular division e.g., DNA replication occurs while the nuclei are at the basal surface and mitosis occurs while the nuclei are at the apical surface [34]. Unlike whole cell movement, nuclear migration has been reported to occur at much greater speeds. For instance, Szabo et al. were able to show that bipolar cell nuclei could move at a rate of up to 100 μm/hr [35].

Irrespective of type and speed of migration, it is important to note that injury-induced nuclear movement was directed toward the injury site. This observation supports the view that Müller glial cells are playing an active role in retinal reorganization, and potentially regeneration, following injury. Transdifferentiation is a process characterized by a loss of Müller cell phenotype, subsequent stem cell marker expression, and generation of retinal neurons [9,13]. We were unable to identify such a process in this study using the injury model of laser photocoagulation. For instance, injury-induced expression of stem and photoreceptor precursor markers, typically found in both newly generated and developing photoreceptors (photoreceptor cells are the initial and primary cell type affected by this type of injury), could not be detected at any of the time points tested in this study. Several explanations as to why we did not observe similar results as those mentioned can be provided, the most obvious being the size and type of retinal injury inflicted. For instance, as demonstrated by Takeda et al. [13], it may be necessary to induce a milder pan-retinal injury, e.g., by using a glutamate analog, α-aminoadipate, to induce photoreceptor toxicity. Unlike laser photocoagulation, glutamate excitotoxicity does not destroy the outer nuclear layer, but rather leaves the photoreceptor layer intact to provide support and inductive signals for potential Müller cell transdifferentiation. It is also possible that the different results seen here are due to species variations. For instance, unlike our experiments on mice, the aforedescribed studies have used rat or rabbit animal models.

In any case, one has to ask whether inducing stem cell properties in mammalian Müller cells is a reasonable approach for retinal repopulation following cell loss. For example, photoreceptors outnumber Müller glia in the mouse retina by a ratio of approximately 30:1 [36]. Thus, for transdifferentiation to be effective in a small laser wound, about 100 Müller cells would have to replace up to 3,000 photoreceptors to completely restore the cell numbers originally found in the normal retina; this is a low estimate since it requires that every single Müller cell in the affected area adopt stem cell capabilities and exclusively generate photoreceptor cells. As shown in recent studies this is not the case: the number of Müller cells capable of transdifferentiation is quite small and far from what would be required for retinal reconstruction. Thus, to provide even slight visual improvements, the number of functioning photoreceptor cells produced from Müller glia would have to be significantly enhanced.

In conclusion, although we have shown that retinal laser injury is capable of stimulating a significant glial reaction within the retina (marked by cellular reactivity, proliferation, and targeted Müller cell nuclear migration), we have not been able to identify the existence of Müller cell transdifferentiation in this model.
